# Core clock regulators in dexamethasone-treated HEK 293T cells at 4 h intervals

**DOI:** 10.1186/s13104-021-05871-7

**Published:** 2022-01-28

**Authors:** Rafailia A. A. Beta, Zoi V. Arsenopoulou, Amalia Kanoura, Dimitrios Dalkidis, Rafaela Avraamidou, Nikolaos A. A. Balatsos

**Affiliations:** Department of Biochemistry and Biotechnology, University of Thessaly, Larissa, Greece

**Keywords:** Circadian rhythms, Cell clock resetting, Core clock regulators

## Abstract

**Objective:**

The study of the circadian clock and its mechanisms is easily facilitated through clock resetting in cell culture. Among the various established synchronizers of the circadian clock in cell culture (temperature, serum shock, glucocorticoids), the artificial glucocorticoid Dexamethasone (DEX) is the most widely used. DEX treatment as a protocol to reset the circadian clock in culture gives simple readout with minimal laboratory requirements. Even though there are many studies regarding clock resetting in culture using DEX, reference points or expression patterns of core clock genes and their protein products are scarce and sometimes contradict other works with similar methodology. We synchronise a cell line of human origin with DEX to be used for studies on circadian rhythms.

**Results:**

We treat HEK 293T cells with DEX and describe the patterns of mRNA and proteins of core clock regulators, while making a clear point on how *CLOCK* is less than an ideal molecule to help monitor rhythms in this cell line.

**Supplementary Information:**

The online version contains supplementary material available at 10.1186/s13104-021-05871-7.

## Introduction

Circadian rhythms synchronise physiology, metabolism and behavior of almost all living organisms. In mammals, the rhythms are regulated by circadian clocks; the master clock or central oscillator is located in the suprachiasmatic nucleus (SCN) of the brain, while molecular mechanisms of the clock reside in all tissues of the body [1]. The circadian clocks are biochemical oscillators that incorporate input signals (zeitgebers or synchronizers) and produce output signals through interlocked transcription/translation feedback loops with a period of around 24 h [2–4]. In the core of these loops reside circadian transcription factors, which drive the initiation of rhythmic transcription of target genes or circadian controlled genes [5], while the periodic degradation of the transcripts and the proteins shape overall rhythmicity. These core transcription factors are negatively regulated by some of the protein products of target genes whose transcription they regulate and vice versa. The main transcription factor complexes in mammals are that of CLOCK:BMAL1 and PER:CRY. More specifically, the two core clock regulators CLOCK and BMAL1 form a pioneer heterodimeric transcription factor that promote rhythmic chromatin remodelling and activate the expression of their transcriptional repressors including *PER1, PER2, CRY1* and *CRY2*, as well as to the transcription initiation of multiple clock controlled genes [5]. After translation and the assembly of the PER:CRY complex, they lead to inhibition of the CLOCK:BMAL1 complex and transcriptional repression of their targets while activating *CLOCK* and BMAL1 transcription contributing to the continuity of the process. Apart from these 2 main complexes, multiple proteins have been associated with the central oscillator and exhibit circadian expression and contribute to the regulation of circadian rhythms [4, 6].

Expression of clock genes has been studied in primary cultures as well as in immortalized cell cultures [7, 8] and resetting the clock in culture by glucocorticoids was described over 20 years ago [9]. Many cell types have been used in studies where the clock has been reset in culture from immortalized cells of human or mouse origin to cancer cells [8]. However, studies where the clock has been reset in culture have sometimes contradicting results regarding the expression patterns of core clock regulators [7, 10–12]. HEK293T cell line derives from human kidney, an organ that maintain internal circadian clock, which is central to renal metabolic and homeostatic processes and is involved in drug disposition [13, 14]. Further, HEK 293T cells are highly transfectable and widely used in recombinant protein production [15]. Several synchronizers of the circadian clock in cultured cells have been used, as temperature, serum shock and glucocorticoids [9, 16, 17]. Among them, the artificial glucocorticoid Dexamethasone (DEX) is perhaps the most widely used. Nevertheless, a study to describe circadian synchronization in HEK293T cells with DEX is still pending.

In this work, we synchronize HEK293T cells with DEX harvested at 4-h intervals over 24 h and analyse mRNA and protein levels of core clock regulators *CLOCK*, *PER2* and *BMAL1* providing pilot results of the pattern of these factors over the time course of the experiment. The results described in this work serve as a reference for future studies that depend on clock resetting in HEK 293T cells, highlighting the validity and applicability of clock resetting in culture with DEX and RT-qPCR of endogenous targets and hopefully it will serve as a reference point for researchers that wish to perform experiments with similar design or in the same cell line.

## Main text

### Materials and methods

#### Cell culture

HEK 293T cells were from ATCC (CRL-3216). All cell culture reagents were purchased from Biosera (Nuaillé, France). HEK 293T cells were cultured in DMEM with 10% fetal bovine serum (FBS), 1 × antibiotic/antimycotic. The cells were cultured at 37 °C, with 5% CO_2_ and were frequently checked for mycoplasma infection by PCR.

#### Clock resetting in culture

Cells were plated in 6 cm culture dishes or 6-well plates and the culture medium was supplemented with 100 nM DEX in PBS (DEX, SIGMA-ALDRICH, MO, USA) for 2 h [18]. The cells were harvested 24 h after DEX treatment at 4-h intervals for 24 h in total. Control conditions were also included; the same number of plates and dishes was plated and treated with 0.004% DMSO in PBS (solvent control) in complete DMEM for 2 h.

#### RT-qPCR and data analysis

Total RNA from HEK 293T cells was isolated using TRI-reagent (SIGMA-ALDRICH, MO, USA) and quantified using NanoDrop 2000c (Thermo Fisher Scientific, MA, USA). For the detection of specific mRNAs, 1 μg of total RNA was used for cDNA synthesis with PrimeScript 1st strand cDNA synthesis kit (TaKaRa Bio, CA, USA). Real-time quantitative PCR was performed with SYBR Select MasterMix (Invitrogen, Thermo Fisher Scientific, MA, USA) in a StepOnePlus Real-Time PCR System (Applied Biosystems, Thermo Fisher Scientific, MA, USA). Each sample was assayed in duplicate. *GAPDH* was used as a reference gene. The primers used for the RT-qPCR analysis are listed in Table 1. Data were fitted using sine wave function of GraphPad Prism version 8 (GraphPad Software, San Diego, CA, USA; www.graphpad.com), as has been described [19]. More specifically, Ct values are normalized for expression to *GAPDH* for both biological replicates. Then, data are imported to GraphPad prism and fitted using the sine wave function. The goodness of fit is assessed by the Shapiro–Wilk normality test and the curves are subsequently subjected to Two-Way ANOVA. The normality test assesses how far from normal (Gaussian) the data are; a data set with p ≤ 0.05 fails the normality test, thus deviates significantly from a normal distribution. Accordingly, a statistically significant data set means that it has failed the normality test [19]. Thus, the data presented in this work, are considered for Two-Way ANOVA if they fail the normality test.

**Table 1:** List of primers used for RT-qPCR

*GAPDH*	FWD	AGAAGGCTGGGGCTCATTTG
	REV	AGGGGCCATCCACAGTCTTC
*CLOCK*	FWD	GGCTGAAAGACGACGAGAAC
	REV	GGTGTTGAGGAAGGGTCTGA
*PER2*	FWD	CTGATTGAAACCCCAGTGCT
	REV	ATGGGTTGATGAAGCTGGAC

#### Western Blot

The cells were lysed in IP150 buffer supplemented with 1 mM DTT, 2 mM EDTA and 1 × Set X protease inhibitor cocktail (539196**,** Sigma Aldrich) as has been previously described [20], sonicated and clarified by centrifugation. Proteins were resolved by SDS-PAGE and transferred onto 0.2 μm PVDF membrane (Porablot; Macherey–Nagel, Germany), membranes were blocked and probed using the following antibodies: BMAL1 (#14020, 1:1000, Cell Signaling Technology), CLOCK (#5157, 1:500, Cell Signaling Technology) and β-ACTIN (A5441, 1:5000, Sigma-Aldrich). β-ACTIN was used as a reference. Western blot images were taken using Uvitec Cambridge chemiluminescence imaging system with the help of Alliance Software (ver. 16.06) (Uvitec Cambridge, Cambridge, UK). Protein bands were normalized using ImageJ [21].

### Results

**Fig. 1:**
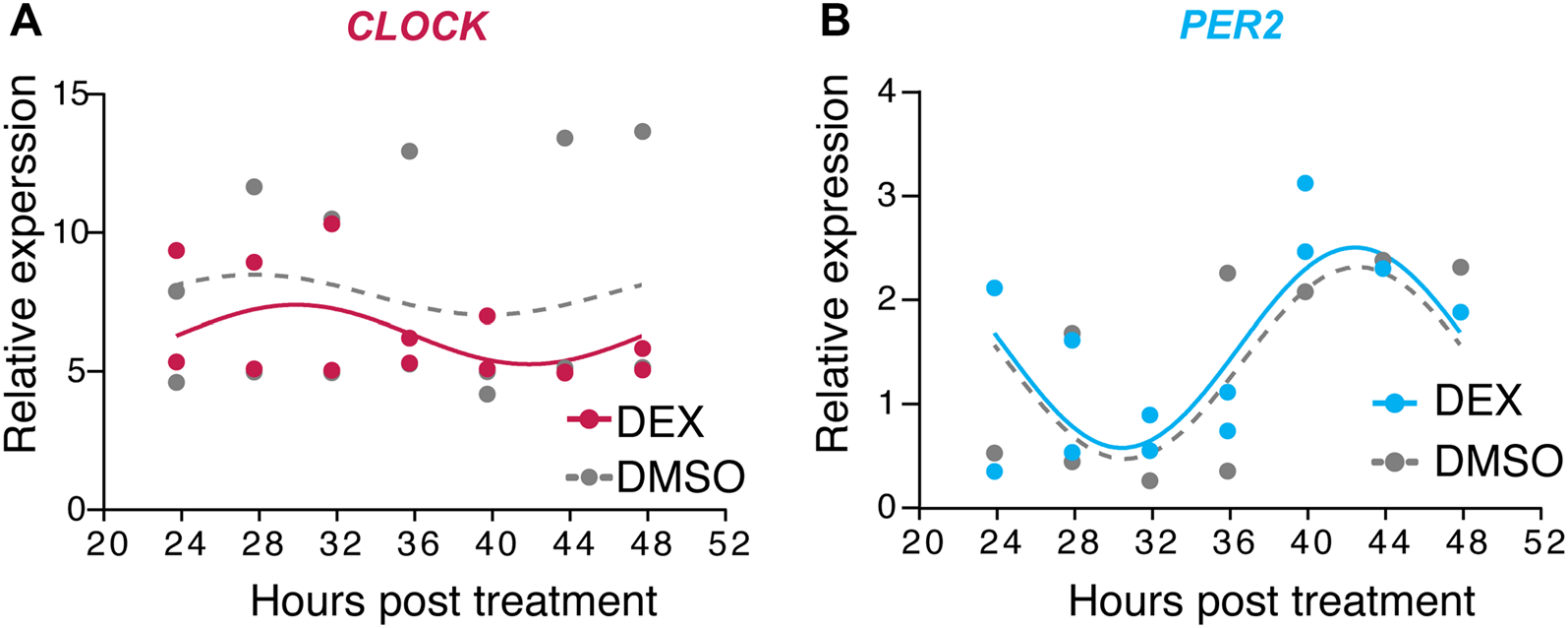
Expression of *CLOCK* and *PER2* in DEX-treated HEK293T cells. **A** Relative *CLOCK* mRNA levels over the time course of 24 h. Fierce red: DEX-treated cells; grey: DMSO-treated cells. **B** Relative *PER2* mRNA levels over the time course of 24 h. Values (n = 2) are normalized for expression with *GAPDH* and plotted. Both replicate experiments are shown on the graphSky blue: DEX-treated cells; grey: DMSO-treated cells

While there are many reports in the literature regarding clock resetting in culture, differences between experimental design and expression patterns of genes and their cognate proteins are often confusing and lead to time delays adding cost to projects as well as adding to the already increased carbon footprint life science research entails [22]. In this work, we present a brief yet comprehensive expression pattern for major circadian regulators in clock-synchronized HEK 293T cells. It is well known that the artificial glucocorticoid DEX is a strong synchronizer for peripheral tissues and cell cultures [23]. Moreover, it has been reported that *CLOCK* is relatively unresponsive to glucocorticoid clock resetting [16]. To this end, we examined *CLOCK* levels after treatment of HEK293T cells with DEX or DMSO (used as DEX’s solvent) over a time course of 24 h (Fig. 1A). Indeed, *CLOCK*’s expression does not seem to be significantly altered in the presence of DEX; the curve fit passes the normality tests and the data were not statistically significant between treatments, thus explaining why *CLOCK* expression is not used in time course experiments where the clock is reset in culture in contrast to other core clock regulators such as *PER*, *CRY*, etc.. Next, we analyzed the expression levels of *PER2*, another major clock regulator (Fig. 1B), known to be under glucocorticoid response element control [16] with overall levels and pattern being in congruence with the literature, even though they differ in the time points of minima and maxima of expression [11, 24]. *PER2* expression is significantly different between DEX and DMSO treatments (p-value = 0.0481), even though they exhibit a similar curve pattern [19].

**Fig. 2:**
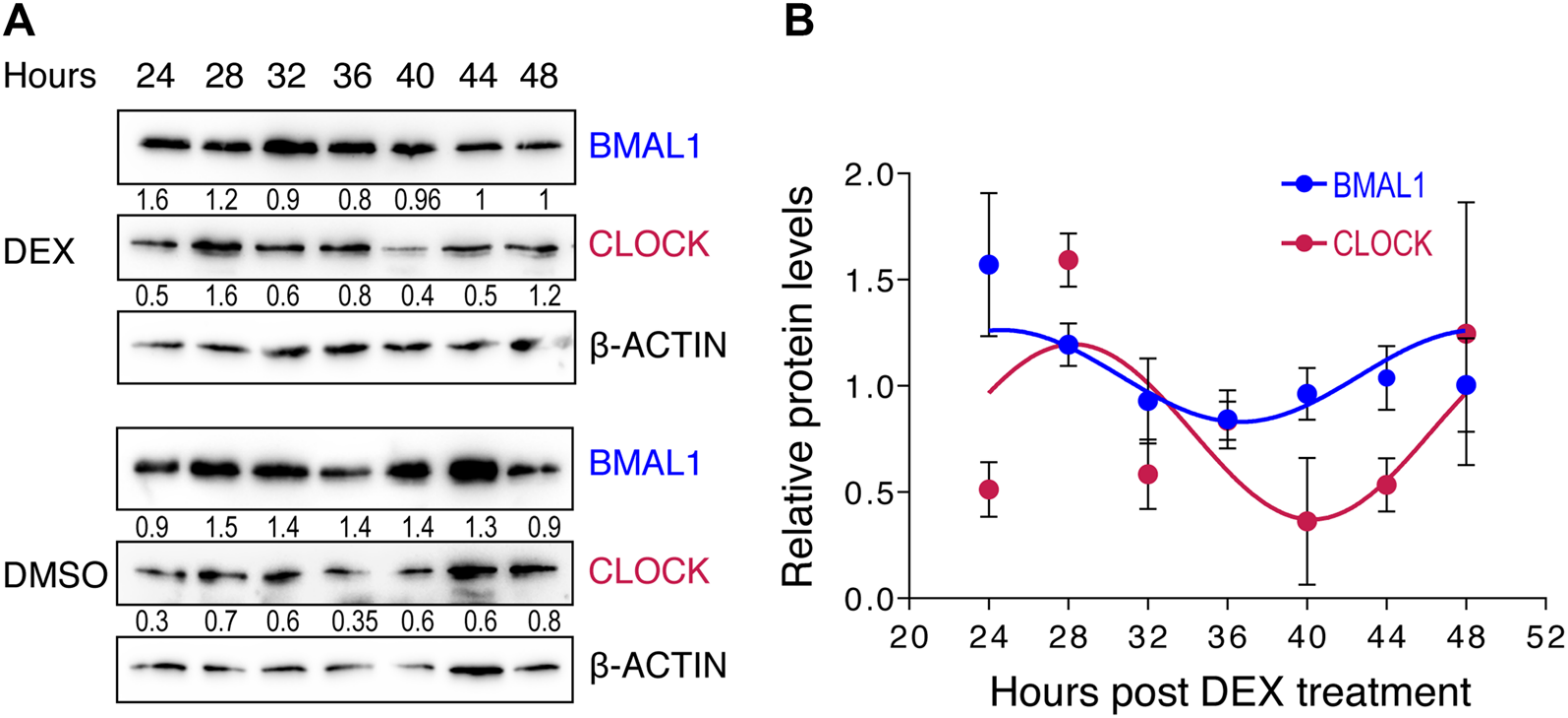
Protein levels of CLOCK and BMAL1 in DEX-treated HEK293T cells. **A** Western blot showing levels of CLOCK and BMAL1. Numbers above the gels indicate time (in hours) post DEX treatment (upper set) or control (DMSO-treated; lower set). β-ACTIN was used as loading control. **B** Levels of CLOCK and BMAL1 in DEX-treated cells after normalization to β-ACTIN. Red and blue lines indicate CLOCK and BMAL1 levels in DEX treated cells, respectively. Values are means ± SEM (n = 3). Numbers below each target indicate protein levels, relative to β-ACTIN. The images are part of the original image of the western blot development. Full-length blots/gels are presented in Additional file 1: Figure S1.

Periodic mRNA abundance stems from rhythmic transcription, stability and degradation, or a combination of these processes that determine overall mRNA life span [4, 25]. Similarly, periodic protein abundance results from both rhythmic translation, often related to periodic mRNA abundance, and protein degradation [25]. Thus, we evaluated protein levels for the two core clock mammalian regulators CLOCK and BMAL1, that comprise a heterodimeric transcription factor. Both proteins are present throughout the time course of the experiment in both DEX-treated and DMSO-treated cells (Fig. 2A) similar to other reports [26, 27]. Plotting of the protein quantification data versus hours post DEX treatment (Fig. 2B), yields a similar pattern for both factors, which is expected considering that the two proteins are part of the same transcription factor complex.

## Limitations

This work is a representation of pilot experiments for the study of the circadian clock in HEK293T cells and has limitations. For example, including different clock resetting methods, such as serum shock and temperature [28], could serve as a more complete description of the data presented here, however it was our choice to include the most commonly used method for clock resetting in culture for all cells expressing glucocorticoid receptor, except SCN cells, which is DEX. The data in this study are representative for HEK293T cells, so discrepancies with other cell models in published works are expected and are discussed in the results section of the Main text.

Moreover, the time intervals selected to present the data in this work are preferred across many similar studies [2, 7, 23, 28]. Shorter time intervals can be studied using stable cell lines expressing core clock regulators fused with bioluminescent reporters. Thus, measurements in very short intervals (e.g. 10 min) have been reported [23], yet such approaches require specific laboratory equipment and produce big data input that is hard and time-consuming to handle. Alternatively, increasing the replicates may improve the statistical analyses, albeit less efficiently than experimenting for shorter time intervals that generally improves temporal resolution [29].

The selection of clock resetting method, choice of the time intervals for data collection and measurements should depend on the type of experiment to be conducted each time so that these experimental factors do not interfere with measurements and data are presented efficiently.

## Supplementary Information

**Additional file 1: Figure S1.** Full size uncropped images of the western blots presented in Fig. 2. Numbers above the gels indicate time (in hours) post DEX treatment (upper set) or control (DMSO-treated; lower set). The dashed line box indicates where the image was cropped in order to be presented in Fig. 2.

## Data Availability

Datasets used during the current study are available from the corresponding author upon reasonable request.

## Abbreviations

SCN: Suprachiasmatic nucleus
DEX: Dexamethasone
FBS: Fetal bovine serum
