# Supplementary figures and images for: Core clock regulators in dexamethasone-treated HEK 293T cells at 4 h intervals
Source: BMC Res Notes. 2022 Jan 28;15:23. doi: 10.1186/s13104-021-05871-7 (PMC8796574; doi:10.1186/s13104-021-05871-7)

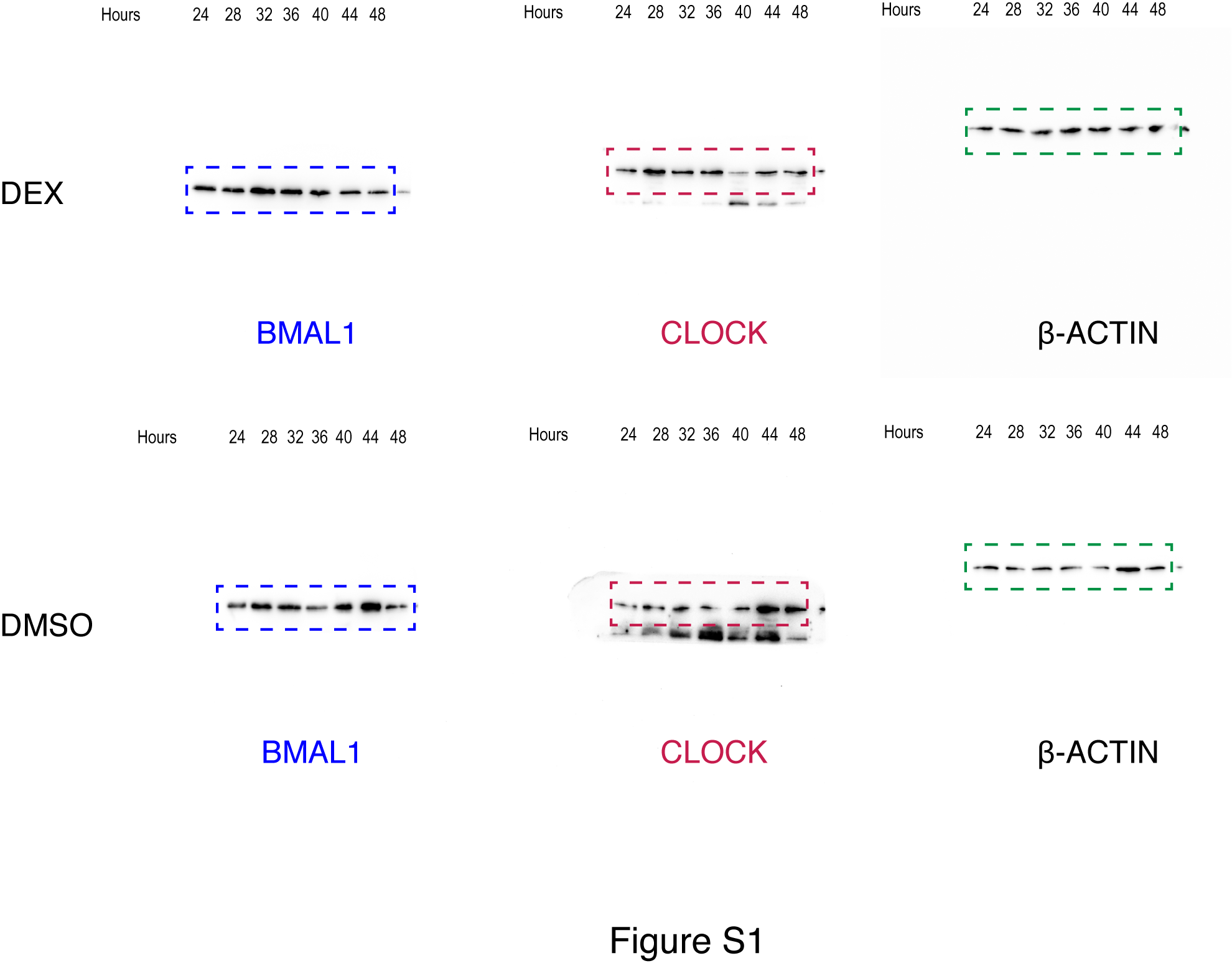

Supplement: Supplementary file 1 — Additional file 1: Figure S1. Full size uncropped images of the western blots presented in Fig. 2. Numbers above the gels indicate time (in hours) post DEX treatment (upper set) or control (DMSO-treated; lower set). The dashed line box indicates where the image was cropped in order to be presented in Fig. 2. [file 13104_2021_5871_MOESM1_ESM.tif]
